# Effects of Physical Therapy on a Patient With Lemierre's Syndrome Who Had Atelectasis and Limited Range of Motion in the Neck

**DOI:** 10.7759/cureus.45533

**Published:** 2023-09-19

**Authors:** Shinichi Onozawa, Fujiko Someya, Masami Yokogawa

**Affiliations:** 1 Rehabilitation Center, Asanogawa General Hospital, Kanazawa, JPN; 2 Department of Physical Therapy, Faculty of Health Sciences, Institute of Medical, Pharmaceutical and Health Sciences, Kanazawa University, Kanazawa, JPN; 3 Faculty of Health and Medical Sciences, Hokuriku University, Kanazawa, JPN

**Keywords:** jugular venous thrombosis, atelectasis, limited range of motion (rom), physical therapy, deep neck abscess, lemierre's syndrome

## Abstract

Lemierre's syndrome (LS) is a severe infectious disease that can lead to the formation of neck abscesses and thrombosis. LS may be an indication for surgery; however, there are few reports on the physical therapy approaches used in patients with LS. A male patient in his 20s reported atelectasis and limited range of motion in the neck after resection of a deep neck abscess on the left side of the neck caused by LS. Thrombophlebitis was also observed around the neck lesion, indicating the risk of pulmonary embolism. Physical therapy was initiated with low-load, deep breathing exercises. Additional breathing exercises, such as respiratory assistance and positive pressure loading, were initiated after the administration of anticoagulants. Although the therapeutic intervention was delayed due to the unstable wound with partially resected muscle, it was assumed that the impairment of the range of motion in the neck was unlikely to persist as the patient was young. No critical adverse events were observed, and the range of motion was recovered such that the patient was able to resume playing baseball. The presence of a venous thrombus and inflammation may affect physical therapy; however, careful management of the exercise load could aid in the safe and effective treatment of LS without the incidence of complications, even in the early postoperative period.

## Introduction

Lemierre's syndrome (LS) is a rare bacterial oropharyngeal infection caused by Fusobacterium necrophorum. LS is characterized by the presence of symptoms such as cervical lymphadenopathy, dysphagia, fever, internal jugular vein thrombosis, and pneumonia [1]. The prevalence of LS among healthy young adults is 3.6 cases per one million individuals, and its mortality rate is estimated to be 4%-12% [2]. Complications of LS include cerebral infarction, lung abscess, and thrombophlebitis [3]. Surgical treatments, such as drainage of abscesses and debridement of tissues, may be required for the treatment of LS [4]. Postoperative limitations in the mobility of the neck have been reported in some cases [5,6]; however, reports of atelectasis have been infrequent [7,8]. The lungs do not expand sufficiently in some patients, resulting in abscess formation and deterioration [7]. Rehabilitation and physical therapy for LS may be performed under disuse conditions, following bed rest during treatment and postoperative functional decline. Although the number of reports on LS has increased in recent years [4], it is considered a forgotten disease [9]. The only study that examined the effect of rehabilitation on LS was a case report pertaining to the field of physical therapy [10], and other case reports on approaches to dysphagia are scattered throughout the literature. No studies have been reported in the field of physical therapy.

Therefore, this case report presents the effects of physical therapy on a patient who developed atelectasis and decreased mobility of the neck after the resection of a deep neck abscess associated with LS. Sufficient risk management plays an essential role in the management of LS. Therefore, this report summarizes postoperative rehabilitation in LS, including risk management and the effectiveness of the intervention, based on a literature review.

## Case presentation

**Figure 1:**
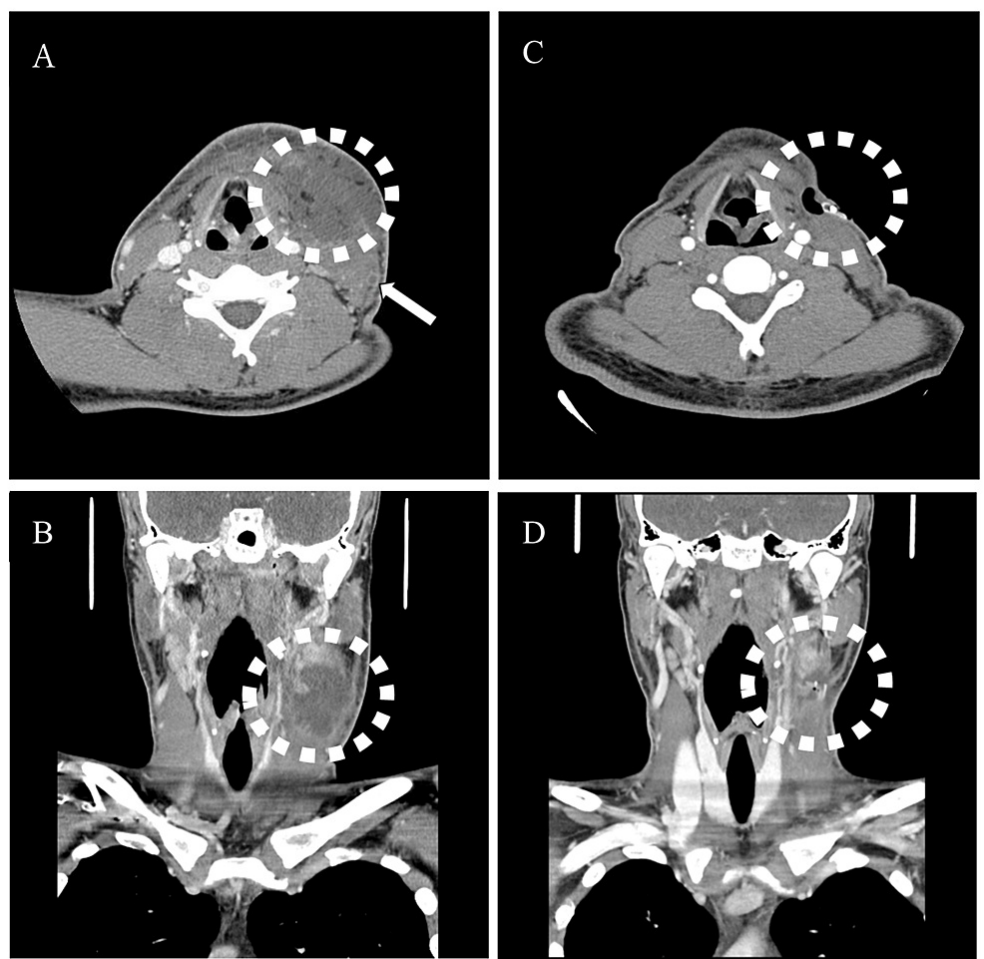
Preoperative (day one) and postoperative (day three) CT images of deep neck abscess and thrombophlebitis in the left external jugular vein. (A) Preoperative CT axial view, (B) Preoperative CT coronal view, (C) Postoperative CT axial view, (D) Postoperative CT coronal view ⇨: Thrombophlebitis of the left external jugular vein

The patient was a male university student in his 20s who was living alone and was a member of a baseball team. He presented to his local physician with a sore throat and fever and was prescribed levofloxacin hydrate, an oral antibiotic (the dosage was not stated on the referral letter and was unknown). As no improvement was observed in his condition after one week, he was referred to an otolaryngologist at our hospital. A neck computed tomography (CT) image revealed a deep neck abscess and thrombophlebitis in the left external jugular vein (Figure 1).

**Figure 2:**
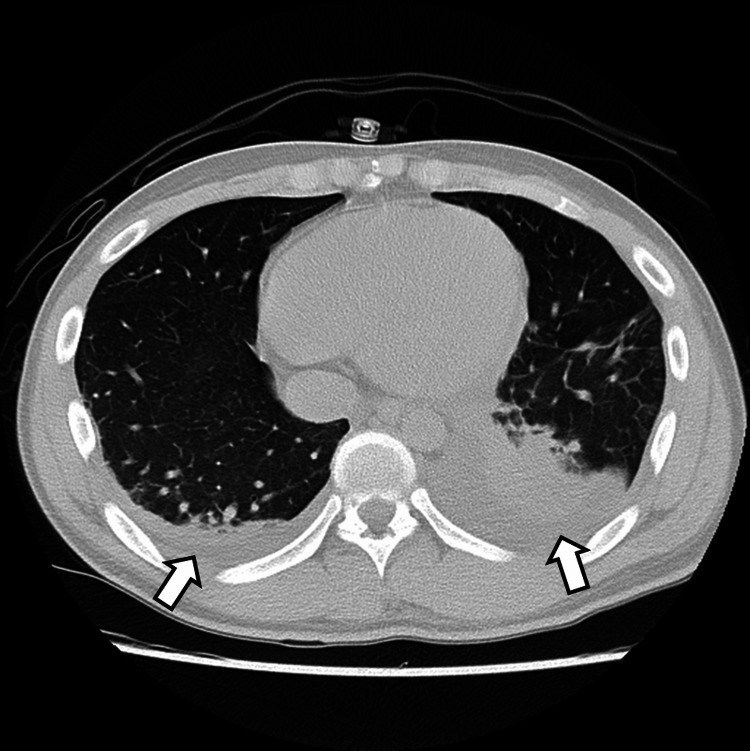
A CT image of the chest on day nine revealed pleural effusion and atelectasis.

Blood examination revealed a high inflammatory response, with a white blood cell count of 326,000/μL and C-reactive protein levels of 24.3 mg/dL. The patient underwent incisional drainage and an excision of the necrotic tissue on day one. The left sternocleidomastoid muscle, platysma muscle, and other subcutaneous tissues with necrotizing fasciitis were partially excised, leaving a Penrose drain. CT performed on day three revealed a residual postoperative abscess (Figure 1); therefore, continuous antibiotic therapy was initiated. Postoperatively, 3 g of sulbactam/ampicillin (SBT/ABPC) was administered four times a day, and 1 g of meropenem hydrate (MPEM) was administered three times a day. Hydrocortisone succinate sodium (200 mg/day) was administered from days 2-4, and the dose was reduced to 100 mg/day by day six. Follow-up CT (Figure 2) performed on day nine revealed pleural effusion in the lungs and atelectasis in the left lung. Therefore, respiratory physical therapy was initiated.

Physical therapy: initial assessment

**Figure 3:**
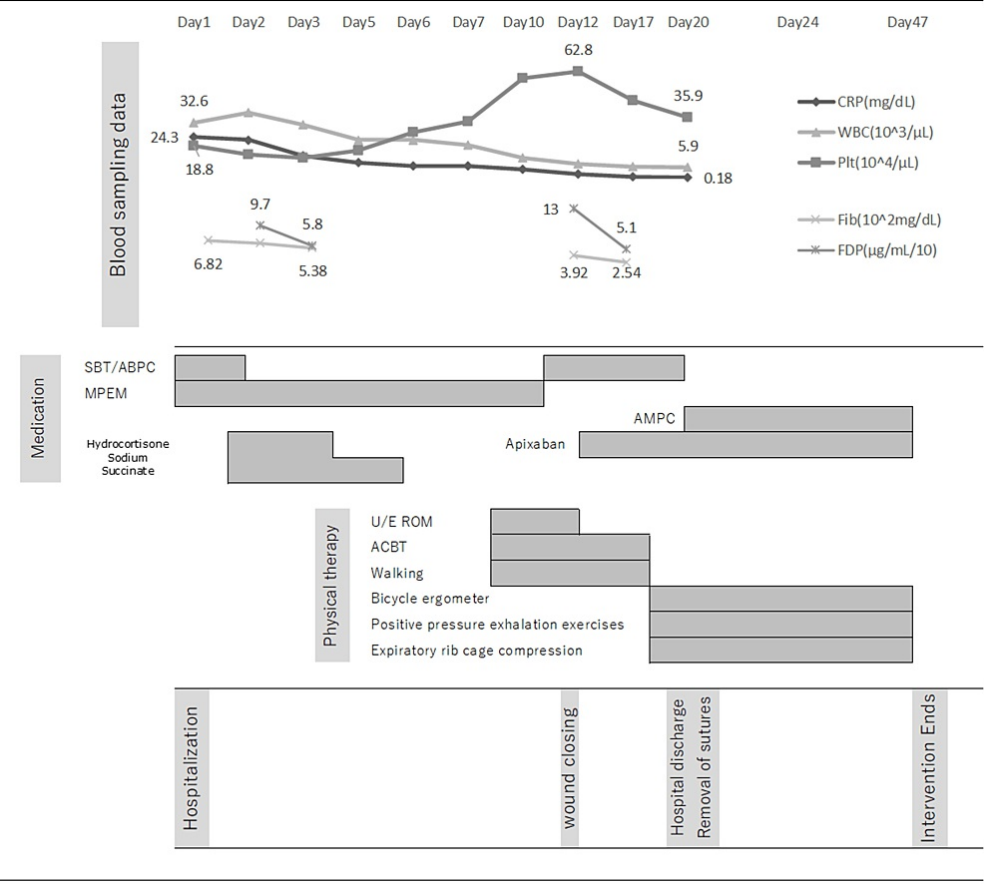
Progress of treatment CRP: C-reactive protein, WBC: White blood cell, Plt: Blood platelet, Fib: Fibrinogen, FDP: Fibrin/fibrinogen degradation products, SBT/ABPC: Sulbactam/Ampicillin, MPEM: Meropenem hydrate, AMPC: Amoxicillin hydrate, U/E ROM: Upper limb range of motion exercises, ACBT: The active cycle breathing technique

The patient was conscious, and his vitals were as follows: blood pressure, 112/68 mmHg; heart rate, 80 beats/min; respiratory rate, 16 breaths/min; and oxygen saturation of the peripheral artery (SpO2), 93% in room air. Respiratory distress was not observed at rest; however, mild shortness of breath was reported while walking along the corridor to the toilet. Bronchial breath sounds were noted in the left dorsal lower lobe, which was the site of atelectasis. Coughing was weak due to wound pain, and swallowing function was limited to four swallows in repetitive saliva swallowing tests [11]. The mouth opening distance was two finger-breadths. The patient’s muscle strength score was 60 (full score) on the Medical Research Council Scale, and there was no obvious limitation in the range of motion (ROM) of the extremities. Neck motion was prohibited during the postoperative period; thus, the ROM could not be measured. Physical therapy was initiated on day nine (Figure 3).

Anticoagulant therapy was not initiated initially for the thrombophlebitis of the left external jugular vein. The active cycle breathing technique (ACBT) [12] was used to treat atelectasis owing to the risk of developing pulmonary embolization. The main technique comprised deep breathing in the sitting position and right and left supine positions. Coughing and huffing were performed weakly to prevent adverse effects on the venous thrombus. Manual rib cage compression was not performed during expiration. Upper-limb ROM and walking exercises were initiated subsequently.

**Figure 4:**
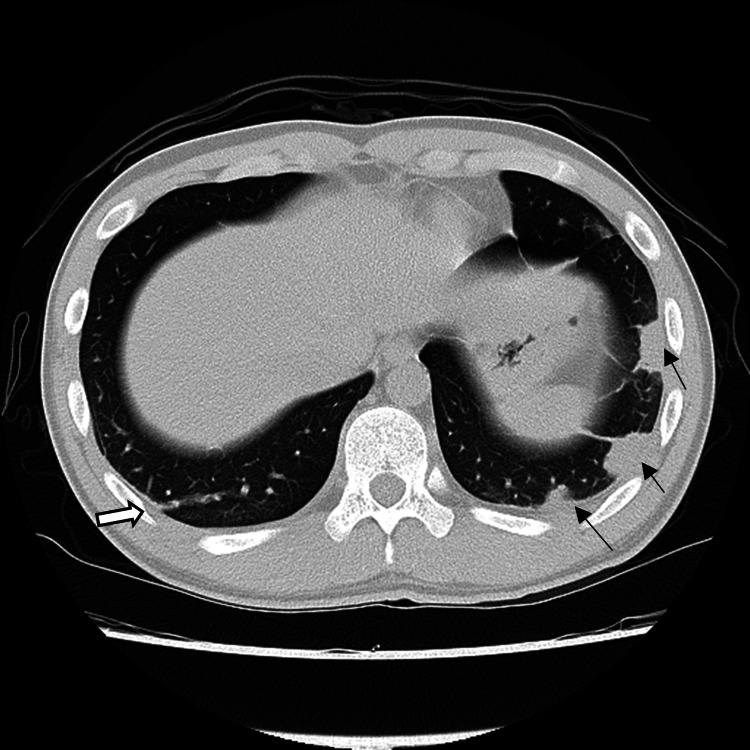
CT on day 18; changes due to septic pulmonary embolus or inflammatory nodules. →: Suspected septic pulmonary embolic changes or encapsulated pleural effusion ⇨: Changes due to septic pulmonary embolism or suspected inflammatory nodule

As the inflammatory response and white blood cell count gradually decreased, MPEM was terminated on day 11, and the administration of 3 g of ABPC/SBT (four times a day) was initiated. The wound was closed following drain removal on day 12, and the administration of 20 mg of apixaban per day was initiated on day 13. Fibrinogen/fibrin degradation products (FDP) and fibrin levels improved from 13.0 to 5.1 (μg/mL/10) and from 3.92 to 2.54 (102 mg/dL), respectively, from days 12-17, and the anticoagulant control was considered to be stable. Positive expiratory pressure exercises using Lung Flute® (ACOUSTIC INNOVATIONS, Japan) and manual rib cage compression during expiration with positional phlegm drainage were performed on day 17 for the treatment of atelectasis. CT performed on day 18 revealed an improvement in atelectasis; however, lesions due to septic pulmonary embolic changes or encapsulated pleural effusion were observed (Figure 4).

**Figure 5:**
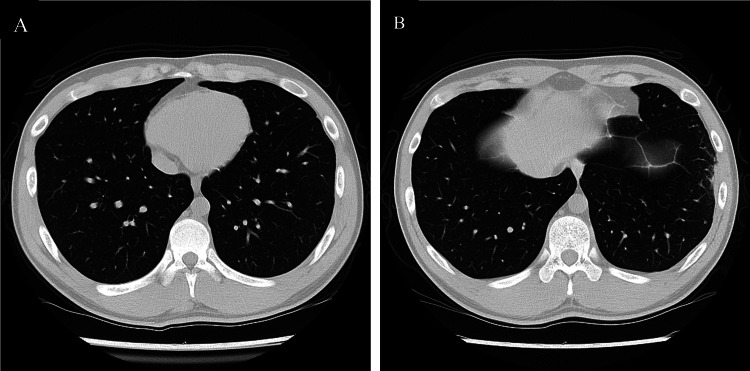
CT on day 46; The hyperabsorption areas in the bilateral lung fields had improved, and the findings of atelectasis and pleural effusion had improved. (A) Areas with pleural effusion or atelectasis were identified on day nine CT. (B) Areas with suspected septic pulmonary embolus, inflammatory nodules, or encapsulated pleural effusion on day 18 CT.

CT revealed a reduction in the size of the cervical abscess on day 19, and the sutures were removed subsequently. Cervical mobilization exercises were performed as the cervical abscess and wound were found to have improved. The ROM of the neck was as follows: flexion, 30°; extension, 60°; right rotation, 30°; left rotation, 45°; right lateral flexion, 20°; and left lateral flexion, 40°. Administration of amoxicillin hydrate was initiated concurrently, and the patient was discharged. Respiratory distress was not observed at rest, and dyspnea and shortness of breath upon exertion were found to have decreased. The resting SpO2 was 98%, and the respiratory sounds were normal. The cough was normal, and dysphagia was not reported. Neck exercises were performed without pain around the wound, and the ROM of the neck increased gradually. The ROM at the final evaluation on day 47 was as follows: neck flexion, 60°; extension, 60°; right rotation, 60°; left rotation, 60°; right lateral flexion, 40°; and left lateral flexion, 40°. Cervical muscle strength was defined as the level of the manual muscle test (MMT) 5 without pain in any direction of movement. No impact on the activities of daily life was observed. Follow-up CT (Figure 5) revealed that the hyperabsorption areas in the bilateral lung fields had disappeared, and the findings of atelectasis and pleural effusion had improved. CT and ultrasonography showed no recurrence of an abscess in the neck, and the patient was able to resume playing baseball at the end of physical therapy.

## Discussion

Physical therapy was hindered by atelectasis and limited ROM of the neck in this case. Respiratory physical therapy for atelectasis generally comprises expiratory pressure exercises and manual breathing assistance [13]. However, the patient had LS-specific thrombophlebitis in the present study, and blood sampling data revealed high FDP and D-dimer levels; therefore, the patient had a high risk of developing pulmonary embolism due to strenuous exercise, such as breath-holding and strong coughing. It has been reported that walking exercise instead of bed rest does not increase the incidence of new pulmonary embolisms, venous thrombus extension is reduced, and pain is reduced if anticoagulation therapy is initiated [14]. Based on these reports, ACBT was administered before anticoagulant control. Therefore, breathing control, deep breathing control, and weak huffing without excessive abdominal pressure elevation were initiated. This approach aimed to minimize the possibility of thrombus release due to an increase in the abdominal pressure associated with forced expiration. ACBT is commonly used to promote airway clearance in individuals with acute and chronic lung diseases accompanied by copious secretions [15]. Westerdahl et al. [16] reported a randomized trial on postoperative deep breathing exercises in patients after coronary artery bypass graft surgery. The results revealed that the atelectasis area in the group performing deep breathing exercises was approximately half that in the non-exercise group. Therefore, positional drainage and deep inhalation of ACBT were initiated in the present case. In addition, huffing and coughing exercises were minimized to avoid excessive abdominal pressure owing to concerns regarding vein thrombosis. Physical therapy did not cause critical adverse events in the present case; however, the safety of exercise in patients with internal jugular vein thrombosis has not yet been verified. Further studies are needed to confirm this hypothesis. High-impact exercises, such as manual breathing assistance, were initiated after anticoagulant control. Thrombophlebitis of the internal jugular vein has been reported in several cases of LS [17,18]. However, Matsunaga et al. [19] reported the incidence of a pulmonary embolism caused by a free thrombus in the external jugular vein. Thus, thromboembolisms, such as pulmonary embolisms, may occur during physical therapy in patients with LS presenting with thrombophlebitis.

As this patient was a baseball player, the residual ROM limitation in the neck had a crucial effect on his quality of life. High inflammation and debridement of the necrotic tissue result in the formation of strong scar contractures in some patients with LS who undergo surgery [5,6]. Surgical resection was also performed in the present case, and the initiation of the ROM exercises was delayed, considering the effects of the residual abscess and wound. There have been no reports of the spread of the residual abscesses owing to exercise; however, the wound was not stable in the present case, and the inflammatory response was high, indicating that early exercise interventions would be risky. Furthermore, partial resection was performed in the present case as the patient was young; therefore, it was hypothesized that sufficient functional recovery could be achieved even after bed rest. As a result, the patient was able to recover neck muscle strength and resume playing baseball without residual limitation of ROM. Several nerves and blood vessels are present within the neck. Consequently, many critical areas of the neck require careful attention [20], necessitating information sharing with the primary surgeon.

## Conclusions

This case report presents the results of physical therapy in a patient with LS who developed atelectasis and decreased mobility of the neck after resection of a deep neck abscess.

The presence of venous thrombus and infection in LS may influence the physical therapy approach, and it is necessary for the physical therapist to understand the pathophysiology and share imaging and laboratory findings with the physician. An appropriate exercise load can improve the symptoms and prevent complications, even in the early postoperative period.
